# Valor Prognóstico do Índice de Carga Inflamatória (IBI) em Eventos Coronarianos

**DOI:** 10.36660/abc.20260174

**Published:** 2026-04-30

**Authors:** Luis Antonio Cavalcanti Matias, Pedro Pereira Tenório

**Affiliations:** 1 Universidade Federal do Vale do São Francisco Paulo Afonso BA Brasil Universidade Federal do Vale do São Francisco (UNIVASF), Paulo Afonso, BA – Brasil

**Keywords:** Infarto do Miocárdio, Placa Aterosclerótica, Tomografia Computadorizada por Raios X

**Prezado Editor**,

Lemos com grande interesse o artigo original intitulado "Placas Vulneráveis Detectadas por TC Cardíaca e Risco de Infarto Agudo do Miocárdio Futuro: Valor Preditivo dos Escores CAC e do Índice de Carga Inflamatória". Gostaríamos de parabenizar os autores, Ömür et al.,^1^ pela investigação oportuna sobre a integração de marcadores anatômicos e inflamatórios na predição de eventos coronarianos agudos.

O estudo aborda uma lacuna crítica na cardiologia preventiva: a transição do foco apenas na "placa vulnerável" para o conceito de "paciente vulnerável", integrando a carga aterosclerótica sistêmica ao estado inflamatório. A aplicação do índice de carga inflamatória (IBI), produto da Proteína C-Reativa (PCR) pela razão neutrófilo-linfócito no contexto da doença arterial coronariana é louvável. Embora o IBI já fosse utilizado em oncologia, sua validação como preditor independente de infarto agudo do miocárdio (IAM) neste estudo amplia o arsenal de biomarcadores acessíveis.

O uso de uma abordagem multifacetada para o monitoramento dos desfechos, incluindo o sistema nacional de registros eletrônicos de saúde (e-Nabiz) e comunicação direta com outras unidades de saúde, confere alta confiabilidade aos dados de seguimento de 3 anos. A demonstração de que a combinação de altos escores de Cálcio Coronário (CAC > 102) e IBI (> 128) eleva significativamente o risco de IAM (OR 1,484) e oferece uma ferramenta prática para a estratificação de risco em pacientes com dor torácica e placas não obstrutivas (< 50% de estenose).

Apesar dos resultados promissores, alguns pontos poderiam ser discutidos ou refinados em pesquisas futuras. Embora o acompanhamento tenha sido sistemático, o *design* retrospectivo limita a capacidade de inferir causalidade direta e pode introduzir vieses de seleção, apesar do grande tamanho da coorte (n=1.235). O IBI foi calculado a partir de amostras de sangue obtidas em um único ponto temporal imediatamente antes da angiotomografia. Como a inflamação é um processo dinâmico, seria valioso discutir se medidas seriadas do IBI poderiam oferecer uma predição ainda mais acurada. Além disso, a ausência de comparação direta com biomarcadores inflamatórios já consolidados, como a PCR de alta sensibilidade isolada ou a interleucina-6, limita a avaliação do real valor incremental do IBI na estratificação de risco cardiovascular.^2^ Adicionalmente, a natureza monocêntrica do estudo e sua realização em um contexto específico de sistema de saúde podem limitar a validade externa dos achados, restringindo sua generalização para populações com diferentes perfis epidemiológicos e assistenciais.³ O estudo conclui que a abordagem facilita estratégias de tratamento mais específicas. Seria enriquecedor ter destacado se a intensidade da terapia com estatinas ou o uso de agentes anti-inflamatórios alvo (como a colchicina) diferiram significativamente entre os modelos propostos durante o seguimento.

Em suma, o trabalho de Ömür et al.^1^ reforça a importância de olharmos além da estenose luminal, integrando a biologia da inflamação à carga de placa calcificada para melhor proteger nossos pacientes.
