# Supplementary material for: Valor Prognóstico do Índice de Carga Inflamatória (IBI) em Eventos Coronarianos
Source: Arq Bras Cardiol. 2026 Apr 30;123(4):e20260174. [Article in Portuguese] doi: 10.36660/abc.20260174 (PMC13155778; doi:10.36660/abc.20260174)
Supplement: Translation

# Translation: Prognostic Value of the Inflammatory Burden Index (IBI) in Coronary Events

**DOI:** 10.36660/abc.20260174i

**Authors:** Luis Antonio Cavalcanti Matias, Pedro Pereira Tenório

**Affiliations:** 1 Paulo Afonso BA Brazil Universidade Federal do Vale do São Francisco (UNIVASF), Paulo Afonso, BA – Brazil

**Keywords:** Myocardial Infarction, Atherosclerotic Plaque, X-Ray Computed Tomography

**Dear Editor**,

We read with great interest the original article entitled "Cardiac CT-Detected Vulnerable Plaques and Risk of Future Acute Myocardial Infarction: Predictive Value of CAC Scores and Inflammatory Burden Index". We want to congratulate the authors, Ömür et al.,^1^ on their timely investigation into the integration of anatomical and inflammatory markers for predicting acute coronary events.

This study addresses a critical gap in preventive cardiology: the shift from focusing solely on the "vulnerable plaque" to the concept of the "vulnerable patient," integrating the systemic atherosclerotic burden with the inflammatory state. The application of the Inflammatory Burden Index (IBI), a product of C-Reactive Protein (CRP) and the neutrophil-to-lymphocyte ratio, in the context of coronary artery disease is commendable. Although the IBI was already used in oncology, its validation as an independent predictor of acute myocardial infarction (AMI) in this study expands the arsenal of accessible biomarkers.

The use of a multifaceted approach to outcome monitoring, including the national electronic health records system (e-Nabiz) and direct communication with other health units, confers high reliability to the 3-year follow-up data. The demonstration that the combination of high Coronary Calcium (CAC > 102) and IBI (> 128) scores significantly increases the risk of AMI (OR 1.484) offers a practical tool for risk stratification in patients with chest pain and non-obstructive plaques (< 50% stenosis).

Despite the promising results, some points could be discussed or refined in future research. Although the follow-up was systematic, the retrospective design limits the ability to infer direct causality and may introduce selection biases, despite the large cohort size (n=1,235). The IBI was calculated from blood samples obtained at a single time point immediately before CT angiography. As inflammation is a dynamic process, it would be valuable to discuss whether serial IBI measurements could offer an even more accurate prediction. Furthermore, the lack of direct comparison with already established inflammatory biomarkers, such as isolated high-sensitivity C-reactive protein or interleukin-6, limits the assessment of the true incremental value of IBI in cardiovascular risk stratification.^2^ Additionally, the single-center nature of the study and its performance within a specific healthcare system context may limit the external validity of the findings, restricting their generalizability to populations with different epidemiological and care profiles.³ The study concludes that the approach facilitates more specific treatment strategies. It would have been enriching to highlight whether the intensity of statin therapy or the use of targeted anti-inflammatory agents (such as colchicine) differed significantly between the proposed models during follow-up.

In summary, the work of Ömür et al.^1^ reinforces the importance of looking beyond luminal stenosis, integrating the biology of inflammation with the burden of calcified plaque to better protect our patients.
