# Supplementary material for: Valor Prognóstico do Índice de Carga Inflamatória (IBI) em Eventos Coronarianos
Source: Arq Bras Cardiol. 2026 Apr 30;123(4):e20260174. [Article in Portuguese] doi: 10.36660/abc.20260174 (PMC13155778; doi:10.36660/abc.20260174)
Supplement: Related texts

# Reply: Carta-resposta

**Authors:** Sefa Erdi Ömür, Emin Koyun, Gökhan Cabri

**Affiliations:** 1 Tokat Gaziosmanpasa Universitesi Tokat Turquia Tokat Gaziosmanpasa Universitesi, Tokat – Turquia; 2 Sivas Cumhuriyet University Sivas Turquia Sivas Cumhuriyet University, Sivas – Turquia

**Prezado Editor**,

Gostaríamos de agradecer sinceramente aos autores pelos seus comentários ponderados e construtivos sobre o nosso artigo intitulado "Placas Vulneráveis Detectadas por Tomografia Computadorizada Cardíaca e o Risco de Futuro Infarto Agudo do Miocárdio: Valor Preditivo dos Escores de CAC e do Índice de Carga Inflamatória". Agradecemos imensamente o interesse demonstrado em nosso trabalho e a valiosa contribuição para o debate em curso nesta área.

Como corretamente enfatizado pelos autores, o objetivo principal do nosso estudo foi ir além do paradigma tradicional da "placa vulnerável" e adotar uma abordagem mais abrangente do "paciente vulnerável", integrando a carga aterosclerótica ao estado inflamatório sistêmico. Essa estrutura integrativa é cada vez mais reconhecida como essencial na predição do risco cardiovascular, dada a função bem estabelecida da inflamação na aterotrombose.^1,2^

Concordamos plenamente com os comentários referentes ao desenho retrospectivo e unicêntrico do nosso estudo, que também foram reconhecidos como limitações em nosso manuscrito. No entanto, gostaríamos de enfatizar que a coorte relativamente grande e a estratégia abrangente de acompanhamento — incorporando registros eletrônicos de saúde nacionais, contato direto com o paciente e verificação cruzada com centros de saúde externos — aumentaram a robustez e a confiabilidade da determinação dos desfechos. Não obstante, estudos prospectivos e multicêntricos são necessários para validar nossos achados em populações mais amplas, particularmente em vista das preocupações com a validade externa.

A observação sobre o uso de uma única medição basal do Índice de Carga Inflamatória (IBI) é altamente relevante. Dada a natureza dinâmica da inflamação, avaliações seriadas de marcadores inflamatórios podem fornecer informações prognósticas adicionais. Estudos futuros que incorporem medições longitudinais de biomarcadores podem, portanto, refinar ainda mais os modelos de predição de risco.

Agradecemos também a sugestão de comparar o IBI com biomarcadores inflamatórios já estabelecidos, como a proteína C-reativa de alta sensibilidade ou a interleucina-6. Embora nosso estudo tenha tido como objetivo avaliar um marcador inflamatório composto simples, acessível e clinicamente aplicável, análises comparativas incluindo biomarcadores já estabelecidos podem ajudar a definir melhor o valor prognóstico incremental do IBI.

Além disso, o comentário sobre as estratégias de tratamento durante o acompanhamento, incluindo a intensidade do uso de estatinas e terapias anti-inflamatórias direcionadas, como a colchicina, é particularmente importante. Avaliar a interação entre a estratificação de risco baseada na inflamação e as intervenções terapêuticas pode fornecer informações valiosas para o manejo personalizado do risco cardiovascular em estudos futuros.

Em conclusão, agradecemos mais uma vez aos autores pelo valioso e construtivo feedback. Acreditamos que esta troca acadêmica contribui significativamente para a compreensão em constante evolução da integração da carga aterosclerótica derivada de exames de imagem e da inflamação sistêmica na avaliação do risco cardiovascular.

## References

[1] 1 Ridker PM, Hennekens CH, Buring JE, Rifai N. C-Reactive Protein and Other Markers of Inflammation in the Prediction of Cardiovascular Disease in Women. N Engl J Med. 2000;342(12):836-43. doi: 10.1056/NEJM200003233421202.10.1056/NEJM20000323342120210733371

[2] 2 Rothwell PM. External Validity of Randomised Controlled Trials: "To Whom do the Results of this Trial Apply?". Lancet. 2005;365(9453):82-93. doi: 10.1016/S0140-6736(04)17670-8.10.1016/S0140-6736(04)17670-815639683

# Reply

**Authors:** Sefa Erdi Ömür, Emin Koyun, Gökhan Cabri

**Affiliations:** 1 Tokat Türkiye Tokat Gaziosmanpasa Universitesi, Tokat – Türkiye; 2 Sivas Türkiye Sivas Cumhuriyet University, Sivas – Türkiye

**Dear Editor**,

We would like to sincerely thank the authors for their thoughtful and constructive comments on our article entitled "Cardiac CT-Detected Vulnerable Plaques and Risk of Future Acute Myocardial Infarction: Predictive Value of CAC Scores and Inflammatory Burden Index" We greatly appreciate their interest in our work and their valuable contribution to the ongoing discussion in this field.

As correctly emphasized by the authors, the primary objective of our study was to move beyond the traditional "vulnerable plaque" paradigm and to adopt a more comprehensive "vulnerable patient" approach by integrating atherosclerotic burden with systemic inflammatory status. This integrative framework is increasingly recognized as essential in cardiovascular risk prediction, given the well-established role of inflammation in atherothrombosis.^1,2^

We fully agree with the comments regarding the retrospective and single-center design of our study, which were also acknowledged as limitations in our manuscript. However, we would like to emphasize that the relatively large cohort and the comprehensive follow-up strategy—incorporating national electronic health records, direct patient contact, and cross-verification with external healthcare centers—enhanced the robustness and reliability of outcome ascertainment. Nevertheless, prospective and multicenter studies are warranted to validate our findings across broader populations, particularly in light of concerns regarding external validity 2.

The observation regarding the use of a single baseline measurement of the Inflammatory Burden Index (IBI) is highly relevant. Given the dynamic nature of inflammation, serial assessments of inflammatory markers may provide additional prognostic information. Future studies incorporating longitudinal biomarker measurements may therefore further refine risk prediction models.

We also appreciate the suggestion to compare IBI with established inflammatory biomarkers such as high-sensitivity C-reactive protein or interleukin-6. While our study aimed to evaluate a simple, accessible, and clinically applicable composite inflammatory marker, comparative analyses including established biomarkers may help to better define the incremental prognostic value of IBI.

Furthermore, the comment regarding treatment strategies during follow-up, including statin intensity and targeted anti-inflammatory therapies such as colchicine, is particularly important. Evaluating the interaction between inflammation-based risk stratification and therapeutic interventions may provide valuable insights for personalized cardiovascular risk management in future studies.

In conclusion, we thank the authors once again for their valuable and constructive feedback. We believe that this academic exchange contributes meaningfully to the evolving understanding of integrating imaging-derived atherosclerotic burden and systemic inflammation in cardiovascular risk assessment.
